# Is cancer a good way to die? A population-based survey among middle-aged and older adults in the United Kingdom

**DOI:** 10.1016/j.ejca.2015.12.018

**Published:** 2016-03

**Authors:** Charlotte Vrinten, Jane Wardle

**Affiliations:** Health Behaviour Research Centre, Department of Epidemiology and Public Health, University College London, Gower Street, London, WC1E 6BT, United Kingdom

**Keywords:** Cancer, Heart disease, Attitude to death, End-of-life, Public perceptions

## Abstract

**Objectives:**

Despite improved outcomes, cancer remains widely feared, often because of its association with a long and protracted death as opposed to the quick death that people associate with that other common cause of adult mortality: heart disease. Former editor-in-chief of the BMJ Richard Smith's view that ‘cancer is the best way to die’ therefore attracted much criticism. We examined middle-aged and older adults' agreement with this view and compared their attitudes towards dying from cancer versus heart disease in terms of which was a good death.

**Methods:**

This study was part of an online survey (February 2015) in a United Kingdom (UK) population sample of 50- to 70-year olds (n = 391), with sampling quotas for gender and education. Five characteristics of ‘a good death’ were selected from the end-of-life literature. Respondents were asked to rate the importance of each characteristic for their own death to ensure their relevance to a population sample and the likelihood of each for death from cancer and heart disease. We also asked whether they agreed with Smith's view.

**Results:**

At least 95% of respondents considered the selected five characteristics important for their own death. Death from cancer was rated as more likely to provide control over what happens (p < 0.001), control over pain and other symptoms (p < 0.01), time to settle affairs (p < 0.001), and time to say goodbye to loved ones (p < 0.001) compared with death from heart disease, but there were no differences in expectation of living independently until death (p > 0.05). Almost half (40%) agreed that cancer is ‘the best way to die’, with no differences by age (p = 0.40), gender (p = 0.85), or education (p = 0.27).

**Conclusion:**

Despite the media commotion, a surprisingly high proportion of middle-aged and older adults viewed cancer as ‘the best way to die’ and rated cancer death as better than heart disease. Given that one in two of us are likely to be diagnosed with cancer, conversations about a good death from cancer may in a small way mitigate fear of cancer. Future research could explore variations by type of cancer or heart disease and by previous experience of these illnesses in others.

## Highlights

• Cancer remains widely feared, because of its link with a long and painful death.
• Many prefer to die from heart disease instead because it is considered ‘quick and neat’.
• However, middle-aged and older adults seem more positive about dying from cancer than heart disease.
• They rated cancer more positively on five characteristics of ‘a good death’.
• In addition, 40% of them viewed cancer as ‘the best way to die’.

*‘Your death, near now, is of an easy sort. So slow a fading out brings no real pain’ Clive James (2014)*.

## 1 Background

Despite improved outcomes over recent decades, cancer remains widely feared, and is frequently seen as synonymous with ‘a death sentence’ [1]. Worse still, cancer death is often portrayed as protracted and painful: *‘[Cancer is] a really unpleasant way to go … I wouldn’t wish it on anyone’* [2], or *‘[There are] much better way[s] to go than lying in a bed for 6 months dying of cancer’* [2]. Fear of cancer death may partly have inspired the so-called ‘war on cancer’ [3], in an effort to reduce the burden of suffering and mortality associated with this disease.

Cancer accounts for nearly a third of all deaths in the United Kingdom (UK) each year [4]. Heart disease claims about the same number of lives every year [4], but public perceptions of what it is like to die from these two diseases are vastly different. Dying from cancer tends to be seen as ‘painful’, ‘dragging on’ and causing extreme suffering, while dying from heart disease is seen as ‘quick and neat’, ‘natural’, and ‘relatively painless’ [2], [5]. These public perceptions influence the dread associated with each disease [2], people’s willingness to engage in health behaviour change to prevent them [2], [5], and research funding allocation [6]. In her classic work ‘Illness as Metaphor’, Susan Sontag states that ‘cancer is more feared than heart disease […] if only because it [i.e. heart disease] can be instantaneous, an easy death’ [7]. In popular opinion, heart disease seems to be ‘the more desirable’ way to go, because of its association with a sudden death [2], [8].

A strikingly contrasting view was expressed by the former editor-in-chief of the BMJ, Dr. Richard Smith, when he argued in a BMJ blog post that cancer is *‘the best way to die’*, because it allows you to *‘say goodbye, reflect on your life, leave last messages, perhaps visit special places for a last time, listen to favourite pieces of music, read loved poems, and prepare, according to your beliefs, to meet your maker or enjoy eternal oblivion’* [8]. His blog post attracted much criticism on Internet forums [8], [9], as well as in the UK's national media [10], [11], [12]. Smith’s views were called *‘insensitive’, ‘misguided’*, and *‘highly offensive’* to cancer patients and their families [10]. Many countered his views by relating stories of people who had died a horrible death from cancer. But is his really such an idiosyncratic view? In the public’s mind, are cancer deaths synonymous with ‘bad deaths’?

What constitutes a good death has been well researched, and various common characteristics of ‘a good death’ have been reported across studies [13], [14], [15]. Examples of these include being in control over what happens, being comfortable (i.e. adequate control of pain and other symptoms), and being afforded dignity and privacy [14], [16], [17]. Most of these studies examine the characteristics of a good death according to patients with a terminal illness, their caregivers, the recently bereaved, or end-of-life healthcare professionals, such as hospice staff. To our knowledge, it is unknown whether the same characteristics are considered important by the general population. In this study, we examined how middle-aged and older adults rated death from cancer and death from heart disease, the two most common types of adult mortality [4], on measures of a good death, to obtain a better understanding of lay perceptions of dying from cancer and to explore whether death from cancer is really perceived as negatively as suggested by the media responses to Smith's blog.

## 2 Methods

This study was part of an online survey on attitudes and beliefs about cancer that we carried out in February 2015 in a UK population sample of 50- to 70-year olds (n = 391), using a commercial sampling service. Quotas were set for gender and education to create equal groups across their categories. Informed consent to participate was obtained at the start of the survey. The study was exempt from ethics approval.

We selected five characteristics of a good death from the end-of-life literature: [13], [14] having control over what happens such as who is present or whether one dies at home or in hospital, having control over pain relief and other symptoms, having the opportunity to settle affairs, having time to say goodbye to loved ones, and being able to live independently and with dignity until death. We asked respondents to estimate the likelihood of each characteristic (four-point scales labelled from 1 ‘extremely unlikely’ to 4 ‘extremely likely’) for death from cancer and death from heart disease. A ‘don’t know’ response option was also provided for these questions. Respondents then rated the importance of each characteristic for their own death (four-point scale from 1 ‘not at all important’ to 4 ‘extremely important’; a ‘prefer not to say’ option was also provided). Finally, we quoted Smith’s argument about cancer being the *‘best way to die’* verbatim [8], and asked respondents whether they agreed with him (‘strongly disagree’, ‘tend to disagree’, ‘tend to agree’, ‘strongly agree’).

Demographic data included gender, education, ethnicity, age, and diagnosis of cancer (excluding non-melanoma skin cancer) and heart disease, which were assessed with simple questions. Age was dichotomised to create younger and older groups (‘50–60’ versus ‘61–70’). Education was classified into three levels: ‘finished education at or before age 15’, ‘completed CSEs or O-levels’, and ‘finished A-levels or higher education’. The ethnicity question had the following response options: ‘White’, ‘Black’, ‘Asian’, ‘other’, and ‘prefer not to say’. ‘Prefer not to say’ and ‘don’t know’ responses were coded as missing throughout.

To assess the validity of our choice of characteristics of ‘a good death’, we looked at the response distributions of the importance of each characteristic for respondents’ own death. We calculated mean scores for the likelihood of each characteristic, as well as across all five items, for death from cancer and heart disease. We used t-tests to examine differences between the scores for cancer and heart disease, both for the combined measure and for individual items. Finally, we determined the percentage agreement with Smith’s statement and examined associations between agreement and ratings of the likelihood of the good death characteristics for cancer. Associations with demographic variables were examined using analyses of variance and chi-square tests as appropriate. When associations with demographic variables were found, these were adjusted for in subsequent analyses. All analyses were done using SPSS v22, and a p-value of <0.05 was defined as threshold for statistical significance.

There are two important points that we decided not to address in this study. Firstly, evidence from the previous studies suggests that public perceptions of cancer vary across different types of cancer, according to their perceived survivability, stigma, visibility, and other characteristics [18], [19]. The same may be true for different types of heart disease. In the present study, we did not distinguish between different types of cancer or heart disease because we were interested in the comparison of generic lay perceptions of ‘cancer’ versus ‘heart disease’. Previous research suggests that these lay perceptions are very different, regardless of the variability within each disease group [2].

Secondly, perceptions of what it would be like to die from a particular disease may be shaped by past experiences of others dying from that disease. However, given that most people have experiences of cancer in others [20] and the high prevalence of cancer and heart disease deaths in the general population, it may be the quality of the past experience (what kind of death someone died, rather than just the experience that someone died) that is important in how illness experience affects illness perceptions. To our knowledge, there are no validated measures for the quality of past illness experiences in others, and for this reason, we decided not to include measures of illness experience in our survey.

## 3 Results

The mean age of our sample was 60.4 years (SD = 6.0). The majority of respondents (98%) were from a White ethnic background; so, differences by ethnicity were not explored further. Consistent with the sampling quotas applied for gender and education, approximately half (52%) the sample were female and 34% had finished school at or before 15 years, while 34% had A levels or higher education (Table 1). Thirty-six respondents (9.2%) were diagnosed with heart disease, and 33 (8.4%) with cancer. Excluding these participants did not change the direction of the results; so, the analyses for the bigger sample are presented below.

**Fig. 1:**
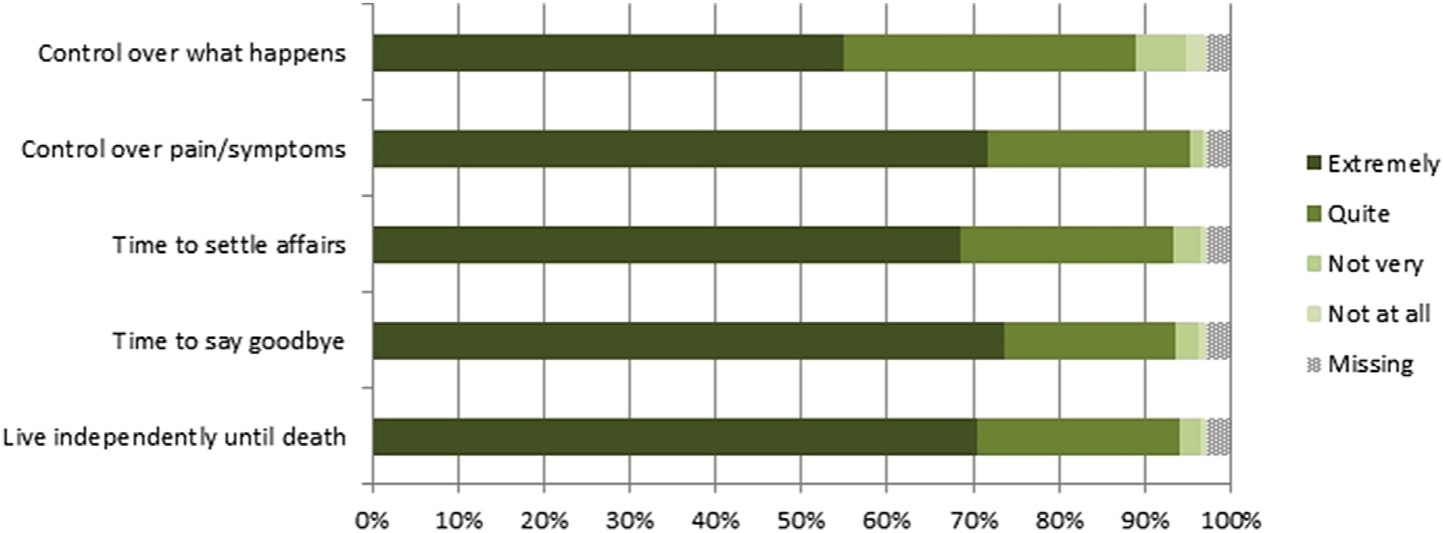
Respondents’ ratings of importance of each characteristic of ‘a good death’ for their own death (N = 391).

Fig. 1 shows respondents’ ratings of the importance of each characteristic of ‘a good death’ for their own death. At least 95% of respondents agreed that these characteristics would be important, with mean ratings for each characteristic between ‘quite’ and ‘extremely’ important. The response distributions were similar across items, except for ‘having control over what happens when you die’, which fewer respondents rated as ‘extremely important’ than the other four characteristics (Fig. 1). There were slight differences by gender and education, but not age (see Table 1); so, we adjusted for demographic variables in the subsequent series of multiple linear regression models run for each characteristic individually (Table 2). These revealed that, on average, scores for women were slightly higher than for men on all but one characteristic, and those with higher levels of education attached slightly more importance to having control over symptoms and having time to settle affairs (Table 1, Table 2). No demographic differences were found for the other characteristics.

**Fig. 2:**
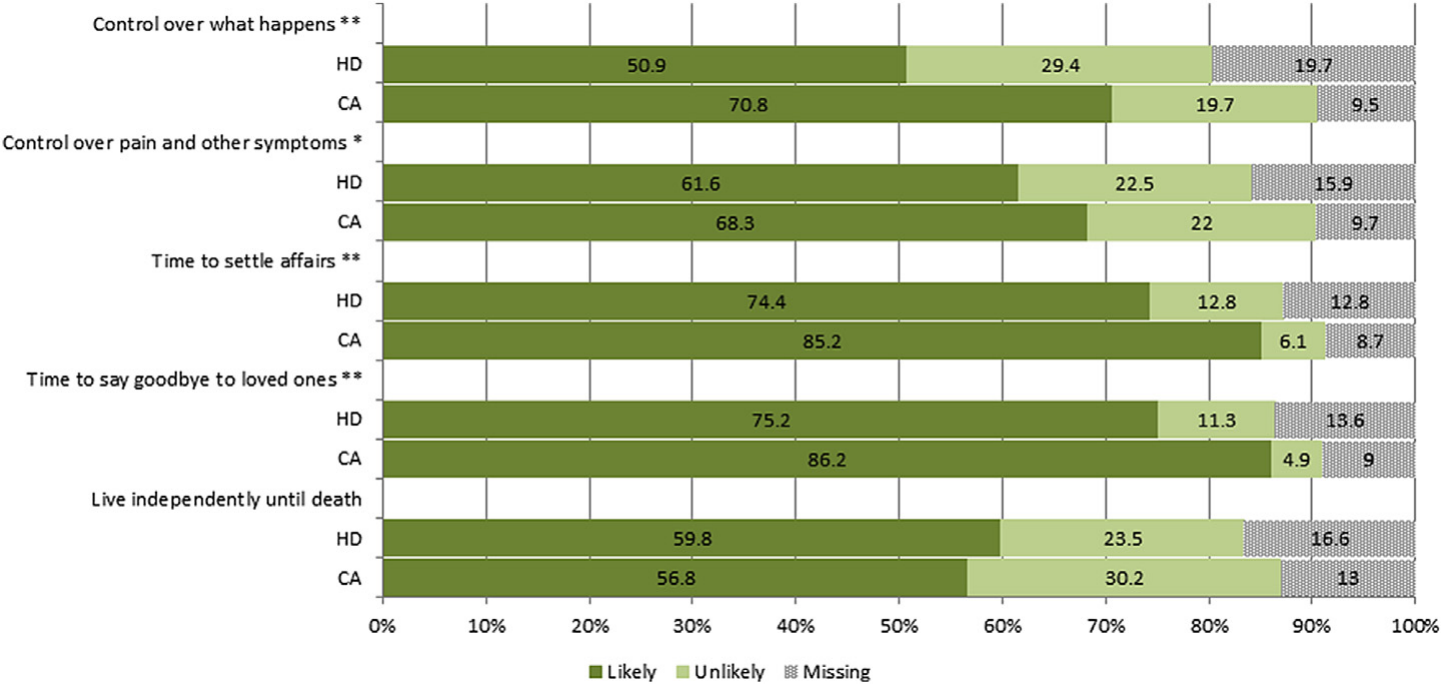
Perceived likelihood of each characteristic of ‘a good death’ for death from cancer (CA) and heart disease (HD); dichotomised for ease of interpretation into ‘likely or very likely’ vs ‘unlikely or very unlikely’. Note: *p < .01, **p < .001.

Comparing the average likelihood ratings for cancer and heart disease across all five characteristics, we found that the average score was significantly more positive for cancer than heart disease death (3.1 versus 2.9, t = 5.02, p < 0.0001), with no demographic differences for either outcome (results not shown). On an individual item basis, death from cancer was seen as significantly more likely to provide control over what happens (3.0 versus 2.8, t = 5.01, p < 0.001), control over pain and other symptoms (3.0 versus 2.9, t = 2.84, p ≤ 0.01), time to settle affairs (3.3 versus 3.1, t = 3.95, p < 0.001), and time to say goodbye to loved ones (3.3 versus 3.1, t = 5.01, p < 0.001, see also Fig. 2). There were no differences in likelihood of living independently until death (p > .05).

When respondents were asked directly whether they agreed with Smith that cancer was *‘the best way to die’*, 40% agreed (34% ‘tend to agree’, 6% ‘strongly agree’), while 60% disagreed (32% ‘tend to disagree’, 28% ‘strongly disagree’). Chi-square tests showed no significant demographic differences (results not shown). Respondents who rated cancer death as having relatively more positive features were slightly, but not significantly, more likely to agree (r = 0.09, p = 0.08).

## 4 Discussion

To our knowledge, this is the first study that examined how middle-aged and older adults rate dying from cancer on end-of-life measures of ‘a good death’ and compared these with lay perceptions of dying from heart disease. Contrary to popular belief or the responses to Smith’s blog, middle-aged and older adults rated death from cancer slightly more positively than death from heart disease on five parameters of ‘a good death’. We also found that a substantial proportion (40%) of survey respondents agreed with Smith’s argument that cancer could be *‘the best way to die’*.

The finding that cancer death was viewed more positively than heart disease death is reflected in studies of quality of care. The National Survey of Bereaved People records bereaved people’s views on the quality of care provided to a friend or relative in the last three months of life in England [21]. This annual survey has shown that over the period 2011–2013, quality of care for those who died from cancer was more often rated as ‘outstanding’ or ‘excellent’ (51%) than death from cardiovascular disease (37–40%) or other causes (40–41%) [21]. Studies about the needs and use of palliative care by cancer and cardiac patients have shown that cardiac patients are less likely than cancer patients to be entered on the palliative care register [22] and that they receive fewer health, social, and palliative care services [23].

The relatively more negative tone about death from cancer in the responses to Smith’s blog than seen in our survey could be explained partly by a ‘strength of feeling’ effect. It is well-known in online product marketing research that people with more extreme views (either satisfied or dissatisfied) are more likely to leave a product review [24]. A similar self-selection may have been at work for the blog respondents, resulting in over-representation of the views of those who disagreed. The current survey may suffer less from this strength of feeling effect (since respondents did not know in advance which questions they would be asked in the survey) and may thus form a better indication of lay perceptions of dying from cancer than the blog responses.

The debate about what constitutes a good death, and whether cancer could fit the bill, is an important one. In a world where one in two of us are likely to be diagnosed with cancer [25], of whom many will die of – or with – cancer, it is encouraging that people are able to consider that a cancer death can be ‘a good death’. It was also interesting to see the consensus on what people valued for their own death; we may have little choice over our ultimate cause of death, but we may have some control over how we die, as reflected in increasing interest in advanced directives and care planning [26], [27].

However, plans for end-of-life care may still not always be clearly communicated. A recent survey found that 83% of Britons believe that people in Britain feel uncomfortable discussing death and dying, and 51% do not know the end-of-life wishes of their partner [28]. Preferences for end-of-life care may also not always be acted upon: over half of Britons (59%) said they would not know how to arrange end-of-life care for themselves or a family member [28], and although 70–80% prefer to die at home, nearly half of all deaths in England occur in hospitals [21], [28]. These statistics show that much remains to be done to ensure that a good death becomes a standard of care for the dying. The Cancer Taskforce’s ambition to increase the number of terminal cancer patients who experience ‘a good death’ [29] and the ‘national choice offer’ [30] aspire to open up dialogues between patients and carers about quality of care at the end of life. Future studies will need to assess whether these proposals are effective.

This study has several limitations. In the sampling, quotas were set for education level and gender, but weighting or propensity scoring were deemed inappropriate for such a small sample size, and this means that the sample may not have been representative of the middle-aged and older UK population. Population representativeness may have been further attenuated by the recruitment method. For example, previous research has found a protective effect of Internet use on health literacy decline in older adults, which could have resulted in a relatively more health-literate sample compared with the general population [31]. The current study would need to be replicated in population-representative samples. Future studies would also be needed to determine whether the results of this study are generalisable beyond the UK setting. For example, attitudes towards dying from cancer may be different in countries with less well-developed palliative care systems. Furthermore, only two causes of death were compared; so, we cannot comment on people’s views about what would be ‘the best way to die’ across all causes of death, although we note that 40% of our sample agreed with Smith that cancer would be the best way to go. Although our results show that middle-aged and older adults do seem to be able to consider that there are some benefits to a cancer death, this may also be true for other conditions that have the characteristics that people valued in a cancer death. Furthermore, as explained before, perceptions of dying from cancer or heart disease may vary across different types of cancer or heart disease, and future studies could explore their influence. In addition, this study did not address the origins of people’s attitudes towards dying from cancer versus heart disease, which may be influenced by factors such as previous experience with the illness in others as explained in the Methods section. Future studies could explore the origins of the differences in attitudes that were found in this study. We found statistical differences in the likelihood of dying a good death from cancer or heart disease, but further research would need to explore whether these differences are meaningful in an everyday setting. To our knowledge, no validated scales exist to measure the general public’s views about a good death, and the current study could be used to inform future studies addressing this issue. We only used five characteristics of a ‘good death’, and although respondents indicated that these were indeed important, there may be other features of a good death that could be important to include in future research, such as preferences for quickness of dying [14]. The question about cancer being ‘the best way to die’ was asked after respondents had rated the likelihood of each death characteristic for heart disease and cancer, which may have resulted in higher rates of agreement – an unintended effect of the order in which the questions were asked in the survey. We believe, however, that the reverse order of the items could have resulted in a greater response bias due to inherent preferences to avoid cognitive dissonance, i.e. a respondent could have rated characteristics of a good death for cancer systematically as more unlikely after first disagreeing with the statement that cancer is a good way to die in order to provide consistent answers. Future work should vary presentation order.

## 5 Conclusion

In conclusion, media responses to Smith’s blog post indicated that almost no one shared his views, but our results suggest he is not alone in considering the possibility that cancer is ‘a good way to die’. His blog was regarded as insensitive, but there may be no easy way to raise the topic. Perhaps, he has initiated a conversation that will help all of us – including those with cancer – to consider the end game. Conversations like this one may help allay fear of dying in general and fear of dying of cancer in particular.

## Authors' contributions

CV and JW conceived of the study and were responsible for data collection. Data analysis was done by CV, with input from JW. Both authors contributed equally to interpretation of the analyses and write-up of the results. Both authors have seen and approved the final version of the manuscript before first submission. Due to JW's death, CV was responsible for any subsequent edits to the manuscript.

## Conflict of interest statement

CV and JW have no competing interests.

## Data sharing statement

The dataset and statistical syntax are available from the first author upon request.

## Figures and Tables

**Table 1:** Sample characteristics and the means and SDs of importance of each characteristic of ‘a good death’ for respondents’ own death, scored on a scale from 1 ‘not at all important’ to 4 ‘extremely important’ (N = 380, missing cases excluded).

	Sample, N (%)	Control over what happens, M (SD)	Control over pain and other symptoms, M (SD)	Time to settle affairs, M (SD)	Time to say goodbye to loved ones, M (SD)	Live independently until death, M (SD)
Age (years)						
60 or younger	188 (49.5)	3.49 (0.74)	3.75 (0.52)	3.64 (0.63)	3.74 (0.57)	3.69 (0.56)
Older than 60	192 (50.5)	3.42 (0.69)	3.68 (0.50)	3.67 (0.52)	3.68 (0.56)	3.68 (0.56)
Gender						
Male	183 (48.2)	3.32 (0.79)	3.65 (0.56)	3.58 (0.64)	3.63 (0.65)	3.64 (0.58)
Female	197 (51.8)	3.58 (0.61)	3.77 (0.46)	3.73 (0.51)	3.79 (0.47)	3.73 (0.54)
Education						
Finished school ≤15 years	129 (33.9)	3.38 (0.73)	3.64 (0.60)	3.54 (0.70)	3.67 (0.64)	3.60 (0.65)
CSEs or O-levels	122 (32.1)	3.46 (0.74)	3.69 (0.50)	3.67 (0.55)	3.72 (0.55)	3.72 (0.47)
A levels or higher	129 (33.9)	3.53 (0.67)	3.81 (0.42)	3.76 (0.45)	3.74 (0.50)	3.74 (0.52)

Abbreviations: CSE, certificate of secondary education; SD, standard deviation.

**Table 2:** Multiple linear regression analyses of predictors of importance of each characteristic of ‘a good death’ for respondents’ own death, adjusted for age, gender, and education (N = 380, missing cases excluded).

	Control over what happens		Control over pain and other symptoms		Time to settle affairs		Time to say goodbye to loved ones		Live independently until death	
B (SE B)	β	B (SE B)	β	B (SE B)	β	B (SE B)	β	B (SE B)	β
Age (years)										
>60 versus ≤60	−0.04 (0.08)	−0.03	−0.05 (0.05)	−0.05	0.07 (0.06)	0.06	−0.05 (0.06)	−0.04	0.02 (0.06)	0.02
Gender										
Female versus male	**0.26 (0.07)**	**0.18**	**0.12 (0.05)**	**0.12**	**0.14 (0.06)**	**0.12**	**0.16 (0.06)**	**0.14**	0.09 (0.06)	0.08
Education										
CSEs or O levels versus not	0.06 (0.09)	0.04	0.03 (0.07)	0.02	**0.15 (0.07)**	**0.12**	0.04 (0.07)	0.03	0.12 (0.07)	0.10
A levels or higher versus not	0.15 (0.09)	0.10	**0.15 (0.06)**	**0.14**	**0.23 (0.07)**	**0.19**	0.07 (0.07)	0.06	0.14 (0.07)	0.12
Constant	**3.28 (0.09)**		**3.62 (0.06)**		**3.42 (0.07)**		**3.62 (0.07)**		**3.54 (0.07)**	
Adjusted R^2^ (model)	**0.032**		**0.025**		**0.032**		0.014		0.008	

Abbreviations: CSE, certificate of secondary education; B = unstandardised regression coefficient; SE = standard error; β = standardised regression coefficient.

Note: Values in bold are statistically significant at p < .05.
